# Differential Immune Landscape of Hepatocellular Carcinoma Suggests Potential role of Macrophages in Hepatocarcinogenesis

**DOI:** 10.12669/pjms.37.3.2973

**Published:** 2021

**Authors:** Yusra Shafique, Muhammad Asif Qureshi, Saeed Khan, Talat Mirza

**Affiliations:** 1 Dr. Yusra Shafique, MBBS, M.Phil. Lecturer of Pathology, Dow International Medical College, Dow University of Health Sciences, Karachi, Pakistan; 2 Prof. Dr. Muhammad Asif Qureshi, MBBS, PhD (Glasgow-UK), Professor of Pathology, MA(IR), Postdoc (Germany), CHPE, Dow International Medical College, Dow University of Health Sciences, Karachi, Pakistan; 3 Prof. Dr. Saeed Khan, MSc, PhD, Postdoc (USA). Professor of Pathology, Dow International Medical College, Dow University of Health Sciences, Karachi, Pakistan; 4 Prof. Dr. Talat Mirza, MBBS, M.Phil., PhD. Dean, Research Ziauddin University, Karachi, Pakistan

**Keywords:** HCC, Immunotherapy, Liver cancer, Tumor microenvironment

## Abstract

**Objectives:**

To investigate immune cell densities in tumor microenvironment of hepatocellular carcinoma.

**Methods:**

This cross-sectional study was conducted during 2017-2019 at the Dow University of Health Sciences Karachi. A total of 42 subsequent patients undergoing liver biopsy/resection and diagnosed with hepatocellular carcinoma were included in the study. Moreover, a total of 10 control tissues were also included. In order to investigate immune cells densities in hepatocellular carcinoma, immunohistochemistry was performed using antibodies including α-MPO(neutrophils), α-CD-68(macrophages), α-CD-3(T-cells), α-CD-20(B-cells), α-CD-4(CD4+ T-cells) and α-CD-8(CD8+ T-cells). Quantification of immune cells/mm^2^ was performed as per the College of American Pathologists’ guidelines. Data were analyzed using SPSS version 21. A p-value of 0.05 was considered significant at all times.

**Results:**

We report significantly increased infiltration of macrophages (mean macrophages= 306.57/mm^2^, p-value <0.05), moderately significant infiltration of neutrophils (p-value=0.06) and B-cells (p-value=0.07) while no significant infiltration of CD4+T-cells (p- value=0.31), and CD8+T-cells (p-value=0.39) in tumour microenvironment of patients with hepatocellular carcinoma.

**Conclusion:**

We provide evidence for increased macrophage infiltration in liver cancer microenvironment suggesting a potential role of these cells in hepatocarcinogenesis.

## INTRODUCTION

Hepatocellular carcinoma (HCC) is the 6^th^ most common cancer amongst all cancers, and 4^th^ major cause of cancer associated deaths globally.^1^ In Pakistan, HCC ranks as 11^th^ most common cancer and 7^th^ most common cause of cancer associated death.^1^ Regional cancer registries in Karachi have recently reported HCC to be in the top ten cancers of Pakistan.^2^

Despite recent advances in HCC diagnostics and therapeutics, treatment options for this tumour are limited. Liver transplantation remains the only curative option for end stage patients but is not available to all HCC patients and is extremely expensive.^3^ Limited availability of tyrosine kinase inhibitors and immune check point inhibitors have debatable efficiency in HCC cure along with emerging list of side effects associated with these drugs.^4^,^5^ Novel therapeutic strategies are therefore being actively investigated to address this high-mortality cancer.

HCC almost always develops upon an inflammatory background – indicating a potential role of inflammation in HCC pathogenesis. There are some data available to suggest increased infiltration of immune cells in HCC microenvironment.^6^ Moreover, increased immune infiltration has been reported early in HCC pathogenesis using *in vivo* model of hepatocarcinogenesis.^7^ We therefore believe that HCC is a promising but largely unfathomed candidate for immunotherapy. Moreover, little is known about the leukocyte complexity, their role and their effective modulation within the microenvironment that favors HCC progression. It is therefore highly relevant to investigate molecular players and potential biomolecules in HCC tumour microenvironment not only to better understand HCC pathogenesis but also to identify molecules of diagnostic and therapeutic significance.

In the study presented herein, we have characterized myeloid and lymphoid immune cell infiltration in well-defined human HCC samples. We report increased macrophage infiltration in HCC tissues suggesting their potential role in hepatocarcinogenesis. This finding qualifies for further exploration in order to identify novel immunotherapeutic target for HCC.

## METHODS

This cross-sectional study was conducted at the Department of Pathology, Dow International Medical College, Dow University of Health Sciences during 2017-2019, after obtaining ethical approval from Institutional Review Board, Ref # Ref no. IRB-459/DUHS/-14. A total of 42 subsequent patients diagnosed with primary HCC were included in the study. Patients with metastatic cancer in liver were excluded. Moreover, we also included 10 controls. These controls included adjacent non-tumour tissues (n=6) and tissues from normal individuals enrolled as liver transplant donors (n=4).

### Immunohistochemistry:

Initially all samples were stained with H&E to establish diagnosis of HCC. Subsequently, immunohistochemistry was performed using antibodies to identify neutrophils, macrophages, T-cells and B-cells in HCC microenvironment.

### Quantification of immune cell densities:

Immune cells were quantified as cells/mm^2^ in HCC tumour microenvironment as per College of American Pathologists (CAP) guidelines as previously described.^8^,^9^ Photomicrographs of representative tumor sections x400 magnification were taken for digital annotation using software Image J. Data were entered and analyzed using SPSS version 21 and p-value of <0.05 was considered statistically significant.

## RESULTS

**Fig.1:**
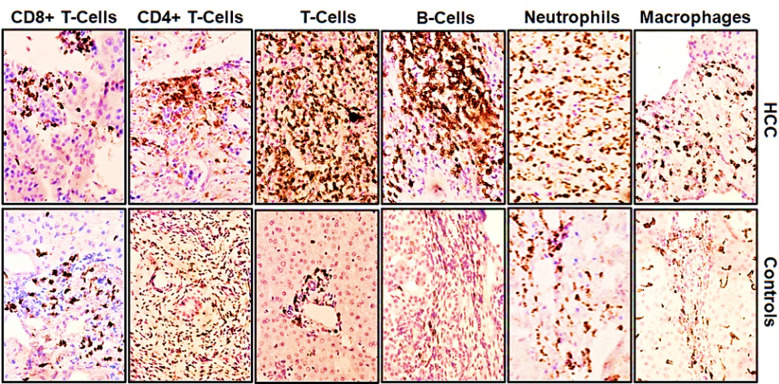
Immune cell infiltration in HCC and control tissues. Microphotographs of original magnification x40 are shown. Immunohistochemistry was performed to stain macrophages, neutrophils, B-cells, T-cells, CD4+ T-cells, CD8+ T-cells using α-CD68, α-MPO, α-CD20, α-CD3, α-CD4 and α-CD8 antibodies respectively.

**Fig.2:**
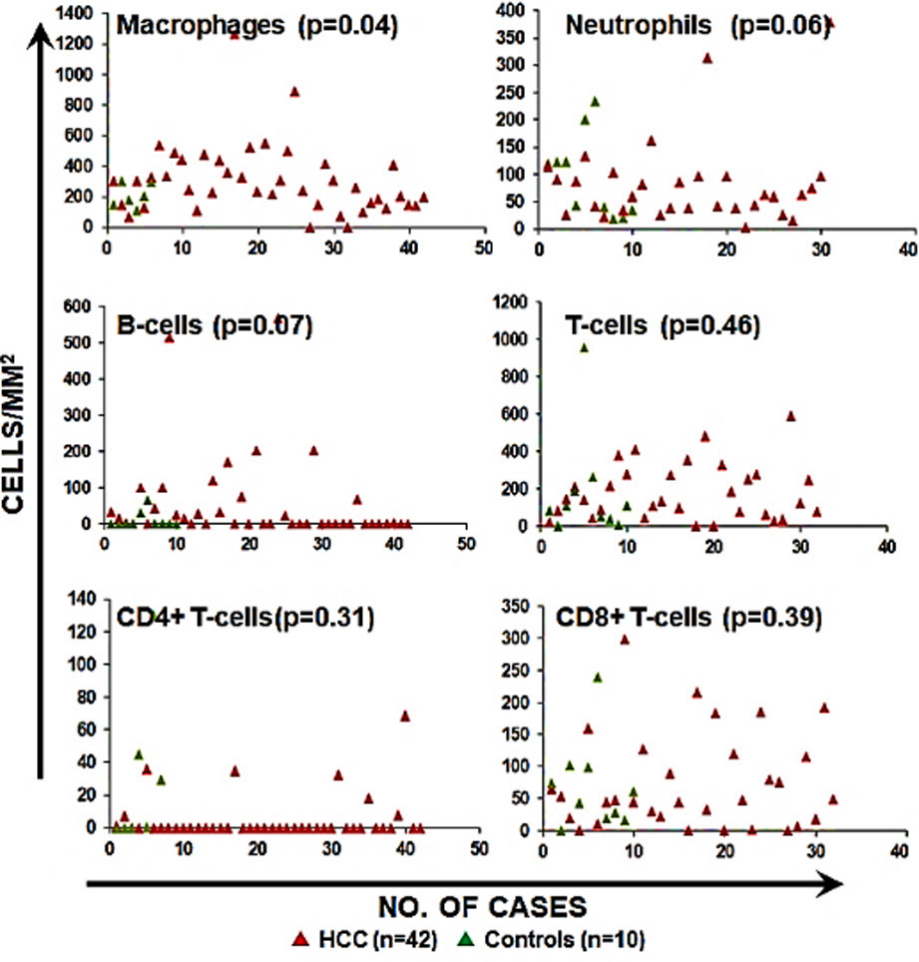
Immune cell densities in in HCC and control tissues. Tumor mapping of immune cell densities as cells/mm^2^ in HCC (n=42) and control tissues (n=10).

Clinico-pathological parameters of HCC tissues are presented in Table-I. In order to investigate any differences in immune cell infiltration patterns between HCC and controls, we used immunohistochemical staining to identify immune cell subsets in HCC and control tissues (Fig.1). Subsequently immune cell densities were plotted and analyzed to investigate if the difference were significantly different (Fig.2).

**Table-I:** Clinicopathological parameters of HCC tissues.

Sample	n (%)
***Hepatocellular Carcinoma Tissues (n=42)***	
Core biopsy samples	29 (69%)
Resection samples	13 (31%)
***Tumor size (n=13)***	
<5cm	08 (62%)
>5cm	05 (38%)
*** LVI (n=42)***	***n***=42
Present	04 (9%)
Absent	38 (91%)
***Tumor Grade (n=42)***	***n***=42
Well differentiated	06 (14%)
Moderately differentiated	34 (81%)
Poorly differentiated	02 (5%)
***Controls (n=10)***	
Donor controls	4 (40%)
Tumor adjacent controls	6 (60%)

We report significantly higher infiltration of macrophage (*p*-value=0.04) in HCC tissues (n=42, mean=306.57 macrophages/mm^2^) as compared to the controls (n=10, mean=205.53 macrophages/mm^2^). Neutrophil infiltration was marginally lower (p-value=0.06) in HCC tissues (n=42, mean=74.96 neutros/mm^2^) compared to the control tissues including biopsy from liver donor and non-adjacent tumour tissues (n=10, mean=140.48 neutros/mm^2^).

Amongst the lymphocytes, there was no significant difference (*p*-value=0.07) in B cell infiltration between controls (n=10, mean=15.82 B-cells/mm^2^) and HCC microenvironment (n=42, mean=55.67 B-cells/mm^2^). Moreover, T-cell infiltration was not different (*p*- value=0.46) between HCC tissues (n=42, mean=154.85 T-cells/mm^2^) and controls (n=10, mean=268.45 T cells/mm^2^). We further investigated if CD4+ and CD8+ T-cells are differentially infiltrated. Our data show that there were no differences in CD4+ and CD8+ T-cells infiltration in HCC compared to the controls.

**Table-II:** Comparison of mean cell densities of immune cells in HCC (n=42) and control (n=10) tissues

Immune cells	Mean immune cells/mm^2^ in HCC	Mean immune cells/mm^2^ in control	p-value
Neutrophils	74.96	140.48	0.06
Macrophages	306.57	205.53	0.04
B-cells	55.67	15.82	0.07
T-cells	154.85	268.45	0.46
CD4+ T-cells	4.91	29.34	0.31
CD8+ T-cells	59.94	92.43	0.39

## DISCUSSION

We show that macrophages infiltration was significantly higher in HCC tissues as compared to the controls. Similar findings have been previously reported using *in-vivo* models as well as human HCC tissues.^10^,^11^ Macrophages play key roles in antigen presentation, tissue remodeling, inflammation regulation, removal of foreign bodies, and tumor progression along with a major role in tumor angiogenesis.^10^-^11^ Cancer cells may express monocyte chemotactic protein CCL2 and M-CSF that recruit mature macrophages into the tumor stroma.^12^ Moreover, increased macrophage densities activate angiogenesis by producing proangiogenic factors including FGF, PDGF, VEGF, MMP-9 amongst others.^13^ Macrophages aid in tumor growth again by increasing angiogenesis by releasing chemokines and growth proliferators such as PDGF, TGF-β, TNF-α, EGF, IL-1.^14^ Macrophages also promote tumour invasion by releasing basement membrane dissolving enzymes including MMP 2, MMP 3, MMP 7 and MMP9. 14 These data are suggestive of a role of macrophages in pathogenesis/progression of tumors (and HCC is no exception to this).

We further report insignificantly lower neutrophils infiltration in HCC tissues as compared to the controls. Rohr-Udilova N et al. reported that there were no significant differences between liver cancer and healthy liver tissues.^15^ On the other hand, Zhou SL et al. and Kuang DM et al. reported significantly increased neutrophil infiltration in HCC tissues in their cohort.^16^,^17^ One of the plausible reasons for increased neutrophil infiltration in thes studies could be release of a pro inflammatory cytokine IL-17 that is generated by activated monocytes/macrophages and its increase in tumoral stroma can cause migration of neutrophils into HCC microenvironment by activating epithelial cells to produce CXC chemokines CXCL5, CXCL8 and CXCL6.^17^ This mechanistic loop (detailing IL-17 axis) was not investigated in our study. Moreover, the infiltrating neutrophils signals a positive feedback loop by producing chemo-attractants (such as IL-8, CXCL1, CXCL2, CXCL5, CXCL6, CXCL7, ENA-78/CXCL5, GCP-2/CXCL6) that recruit more neutrophils to tumor areas and increase their numbers.^18^ Nevertheless, there are only feeble data available on neutrophil infiltration in well-designed HCC studies.

We found that while B cells were infiltrated in the HCC tissues, the difference was not significant when compared to the controls. These findings are in contrast to some of the published studies. For example, Garnelo M et al. reported that in their cohort B cells were heavily infiltrated in HCC.^19^ These findings were similar to Rohr-Udilova N et al.^15^ Cytokines such as IL-2, IL-4, TNF-α, IL-6, IL-10, and INF-γ that are involved in B lymphocyte development can be altered in HCC patients especially with HBV infection. Naïve B cells bind to and are activated by HBV capsid and get stimulated.^20^ Nevertheless, it is important to note that the p-value in our dataset for B-cell infiltration is 0.07, i.e. very close to the traditional significance value of 0.05. It is therefore highly plausible to hypothesize that by increasing the sample size, we may begin to observe significant infiltration of B cells in HCC microenvironment. It is therefore an important area to be explored in future studies.

We found that T-cells were insignificantly infiltrated in HCC tissues in comparison to the controls. These findings are in line with many reports available. For example, Yarchoan et al. reported that the mean density of T cells was decreased in liver microenvironment than normal liver tissue.^21^ It is important to note that various lymphocyte subtype may have different effects on HCC. For example, infiltration of CD8+ T-cells, NK cells and Tregs are associated with patients’ survival.^21^ Moreover, Tregs are known to regulate infiltration of CD8+T cells in tumor microenvironment.^22^ It is therefore important to further investigate lymphocyte subtype infiltrating within HCC microenvironment.

Taken together, we propose a role of macrophages in HCC pathogenesis/ progression. Our findings highlight important players in HCC pathogenesis and thus demand further investigation using larger cohort of samples and further investigating relevant molecular players.

### Limitations of the study:

In this study, we did not perform subtype analyses of T-cell and macrophages to further dissect these immune cell types in hepatocarcinogenesis. Tregs (CD4+CD25+FoxP3+) for example heavily infiltrate HCC tissues^23^ and could be explored further. Similarly, classical and alternatively activated macrophages may be present in HCC tumor *mileu* with differential roles.

## CONCLUSIONS

We provide evidence for increased macrophage infiltration in HCC microenvironment suggesting a potential role of these cells in hepatocarcinogenesis.

### Recommendation:

Modulation of macrophages in HCC microenvironment to investigate their role in tumourigesis is a highly relevant next step. Moreover, subtype analyses of macrophages and T-cells should be performed to better understand immune circuitry involved in HCC progression.

### Authors’ Contribution:

**YS:** Conducted experiments, prepared and analyzed results, manuscript drafting and proof reading.

**AQ:** Conception of idea, execution and management of the whole project. Data indexation, analyses, manuscript drafting and proof reading. As the team lead, also responsible and accountable for the accuracy or integrity of the work.

**SK:** Data analyses, manuscript drafting and proof reading of the manuscript**.**

**TM:** Data indexation, drafting and proof reading of manuscript. Supervision of HCC tissues’ histopathology.

All authors read and agreed to the final version of the manuscript.
